# Determination of the factors responsible for the tropism of SARS-CoV-2-related bat coronaviruses to *Rhinolophus* bat ACE2

**DOI:** 10.1128/jvi.00990-23

**Published:** 2023-09-19

**Authors:** Shigeru Fujita, Yusuke Kosugi, Izumi Kimura, Kenzo Tokunaga, The Genotype to Phenotype Japan (G2P-Japan) Consortium, Keita Matsuno, Jumpei Ito, Kei Sato

**Affiliations:** 1 Hokkaido University, Sapporo, Japan; 2 Tokyo Metropolitan Institute of Public Health, Shinjuku City, Japan; 3 Tokai University, Shibuya City, Japan; 4 Kyoto University, Kyoto, Japan; 5 Hiroshima University, Hiroshima, Japan; 6 Kyushu University, Fukuoka, Japan; 7 Kumamoto University, Kumamoto, Japan; 8 University of Miyazaki, Miyazaki, Japan; 1 Division of Systems Virology, Department of Microbiology and Immunology, The Institute of Medical Science, The University of Tokyo, Tokyo, Japan; 2 Graduate School of Medicine, The University of Tokyo, Tokyo, Japan; 3 Department of Pathology, National Institute of Infectious Diseases, Tokyo, Japan; 4 International Research Center for Infectious Diseases, The Institute of Medical Science, The University of Tokyo, Tokyo, Japan; 5 International Vaccine Design Center, The Institute of Medical Science, The University of Tokyo, Tokyo, Japan; 6 Graduate School of Frontier Sciences, The University of Tokyo, Kashiwa, Japan; 7 Collaboration Unit for Infection, Joint Research Center for Human Retrovirus infection, Kumamoto University, Kumamoto, Japan; 8 CREST, Japan Science and Technology Agency, Kawaguchi, Japan; Loyola University Chicago, Maywood, Illinois, USA

**Keywords:** *Rhinolophus* bat, ACE2, SARS-CoV-2, coronavirus, spike

## Abstract

Differences in host angiotensin converting enzyme 2 (ACE2) genes may affect the host range of SARS-CoV-2-related coronaviruses (SC2r-CoVs) and further determine the tropism of host ACE2 for the infection receptor. However, the factor(s) responsible for determining the host tropism of SC2r-CoVs, which may in part be determined by the tropism of host ACE2 usage, remains unclear. Here, we use the pseudoviruses with the spike proteins of two Laotian SC2r-CoVs, BANAL-20-236 and BANAL-20-52, and the cells expressing ACE2 proteins of eight different *Rhinolophus* bat species to show that these two spikes have different tropisms for *Rhinolophus* bat ACE2. Through structural analysis and cell culture experiments, we demonstrate that this tropism is determined by residue 493 of the spike and residues 31 and 35 of ACE2. Our results suggest that SC2r-CoVs exhibit differential ACE2 tropism, which may be driven by adaptation to different *Rhinolophus* bat ACE2 proteins.

**IMPORTANCE:**

The efficiency of infection receptor use is the first step in determining the species tropism of viruses. After the coronavirus disease 2019 pandemic, a number of SARS-CoV-2-related coronaviruses (SC2r-CoVs) were identified in *Rhinolophus* bats, and some of them can use human angiotensin converting enzyme 2 (ACE2) for the infection receptor without acquiring additional mutations. This means that the potential of certain SC2r-CoVs to cause spillover from bats to humans is "off-the-shelf." However, both SC2r-CoVs and *Rhinolophus* bat species are highly diversified, and the host tropism of SC2r-CoVs remains unclear. Here, we focus on two Laotian SC2r-CoVs, BANAL-20-236 and BANAL-20-52, and determine how the tropism of SC2r-CoVs to *Rhinolophus* bat ACE2 is determined at the amino acid resolution level.

## INTRODUCTION

A series of SARS-CoV-2-related coronaviruses (SC2r-CoVs), which are phylogenetically related to SARS-CoV-2, were identified in *Rhinolophus* bats in China and Southeast Asian countries. For example, RaTG13 (*Rhinolophus affinis* in China, 2013) (1), RmYN02 (*Rhinolophus malayanus* in China, 2019) (2), RacCS203 (*Rhinolophus acuminatus* in Thailand) (3), RpYN06 (*Rhinolophus pusillus* in China, 2020) (4), RshSTT182 (*Rhinolophus shameli* in Cambodia, 2010) (5), BANAL-20-236 (B236; *Rhinolophus marshalli* in Laos, 2020) (6), and Rc-o319 (*Rhinolophus cornutus* in Japan, 2013) (7) were reported so far. Also, SC2r-CoVs, such as Pangolin-CoV and MpCoV-GX, were identified in pangolins (8, 9). These findings support the concept that SARS-CoV-2 emergence is caused by the spillover of certain SC2r-CoV into humans. Importantly, the spike (S) proteins of some SC2r-CoVs, such as RaTG13 (10), B236 (6), and those identified in pangolins (11, 12), are capable of binding to human angiotensin converting enzyme 2 (ACE2), the receptor for SARS-CoV-2 infection. Therefore, it is plausible to assume that some SC2r-CoVs, which can bind to human ACE2, circulate in *Rhinolophus* bats and pangolins in the wild, particularly those residing in Southeast Asian countries. However, because bat *ACE2* genes are highly diversified (13 – 15), it is hypothesized that the differences in bat *ACE2* genes can affect the host range of SC2r-CoVs and further modulate the tropism of host ACE2 for the infection receptor.

B236 is a replication-competent SC2r-CoV that was isolated from rectal swabs of Laotian *R. marshalli* by Temmam et al. (6) In this previous study, the viral sequences of BANAL-20-52 (B52) and BANAL-20-103 (B103) were identified from the samples of *R. malayanus* and *R. pusillus* (6). Importantly, these three BANAL-20 viruses are phylogenetically close to SARS-CoV-2 (6). The amino acid sequences of the S receptor binding motif of B52 and B103 are identical, and the receptor binding domain (RBD) of B52/103 RBD more strongly binds to human ACE2 than that of SARS-CoV-2 S RBD (6). These observations suggest that B236, B52, and B103 are capable of using human ACE2 for the infection receptor; however, the host tropism of these viruses, which can be determined in part by host ACE2 usage, remains unclear. In this study, we particularly focus on the two Laotian SC2r-CoVs, B236 and B52, and elucidate the difference in host ACE2 tropism.

## RESULTS

### Difference in ACE2 tropism between B236 and B52

**FIG 1:**
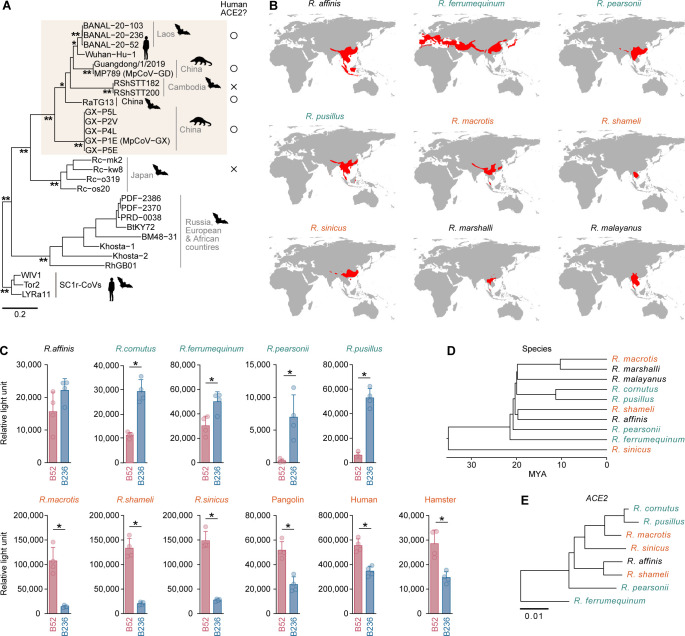
Different ACE2 tropism of the two Laotian SC2r-CoVs. (**A**) Maximum likelihood tree of SC2r-CoVs and SARS-CoV-2 (strain Wuhan-Hu-1) based on their nucleotide sequences corresponding to RBD in S. SARS-CoV-1 (strain Tor2) and two SC1r-CoVs (WIV1 and LYRa11) are included as an outgroup. *, >0.8 bootstrap value; **, >0.9 bootstrap value. The scale bar indicates genetic distance. The usability of human ACE2 for SC2r-CoV infection is indicated with ○ (yes) or × (no), respectively, and the clade of SC2r-CoVs that can use human ACE2 is shaded in brown. (**B**) Geological distributions of *Rhinolophus* bat species. The habitat information originates from the IUCN Red List of Threatened Species website (https://www.iucnredlist.org/). Note that habitat information for *R. cornutus* is not available. Also, the habitat information for *R. marshalli* (B236 was isolated) and *R. malayanus* (B52 was isolated) is included. (**C**) Pseudovirus assay. HIV-1-based reporter viruses pseudotyped with the S proteins of B52 or B236 were prepared. The pseudoviruses were inoculated into a series of HOS-TMPRSS2 cells stably expressing *Rhinolophus* bat ACE2 cells at 1 ng HIV-1 p24 antigen. The infectivity (relative light unit) in each target cell is shown. The host species in which ACE2 is preferred by B52 or B236 are indicated in green and orange, respectively. Data are expressed as the mean with SD. Assays were performed in quadruplicate. Statistically significant differences (*P < 0.05) between B52 and B236 were determined by a two-sided Student’s *t*-test. (**D and E**) Phylogenetic relationship of *Rhinolophus* bat species. (**D**) Time-calibrated species tree for *Rhinolophus* bat species generated by TimeTree5 (16). MYA, million years ago. (**E**) Maximum likelihood tree of *Rhinolophus* bat *ACE2* sequences. The scale bar indicates genetic distance.

We set out to understand the phylogenetic relationship of SC2r-CoVs in *Rhinolophus* bats and pangolins. Consistent with a previous report (6), most of the SC2r-CoVs that are capable of using human ACE2 for the infection receptor formed a cluster with SARS-CoV-2 (Fig. 1A).

To investigate the host ACE2 tropism of two Laotian SC2r-CoVs, B236 and B52, we prepared the HOS-TMPRSS2 cell lines that stably express the ACE2 proteins of eight *Rhinolophus* bat species: *R. affinis*, *R. cornutus*, *R. ferrumequinum*, *R. macrotis*, *R. pearsonii*, *R. pusillus*, *R. shameli*, and *R. sinicus*, most of which are found throughout Southeast and East Asia (Fig. 1B). As controls, we also prepared HOS-TMPRSS2 cells stably expressing the ACE2 proteins of humans, hamsters, and pangolins. We then prepared the human immunodeficiency virus type 1 (HIV-1)-based pseudoviruses with the S proteins of B236 and B52 and inoculated them into a series of target cells. As shown in Fig. 1C, the HOS-TMPRSS2 cells expressing *R. affinis* ACE2 exhibited similar infectivity to both B236 and B52. On the other hand, B236 exhibited higher infectivity than B52 in the cells expressing the ACE2 proteins of *R. cornutus*, *R. ferrumequinum*, *R. pearsonii*, and *R. pusillus* (Fig. 1C). In contrast, B52 exhibited higher infectivity than B236 in the cells expressing the ACE2 proteins of *R. macrotis*, *R. shameli*, and *R. sinicus* as well as those expressing the ACE2 proteins of pangolins, humans, and hamsters (Fig. 1C). These results suggest that the ACE2 tropism of B236 and B52 is different among animal species.

### Interspecies polymorphisms of ACE2 linked to susceptibility to B52 than B236 infection

We next investigated the determinant factor(s) that are responsible for the ACE2 tropism of B52 and B236 on both sides of hosts (i.e., ACE2) and viruses (i.e., viral S). We first addressed the host side and assumed the evolutionary relationship of horseshoe bat species. However, there is no clear correlation between the tropism of B236/52 and the phylogenetic relationship of the host (Fig. 1D). Also, the phylogenetic tree of the ACE2 gene did not show a clear association with the tropism of B236/52 (Fig. 1E). For example, the *R. macrotis* ACE2 gene is phylogenetically closely related to *R. pusillus* and *R. cornutus* (Fig. 1E). However, *R. macrotis* ACE2 showed higher susceptibility to B52 than to B236, while the ACE2 proteins of *R. pusillus* and *R. cornutus* displayed higher susceptibility to B236 than to B52 (Fig. 1C). These observations suggest that the differences in susceptibility of ACE2 proteins to B52 and B236 could not solely be explained by the phylogenetic relationships of the host species and the ACE2 gene (Fig. 1D and E).

**FIG 2:**
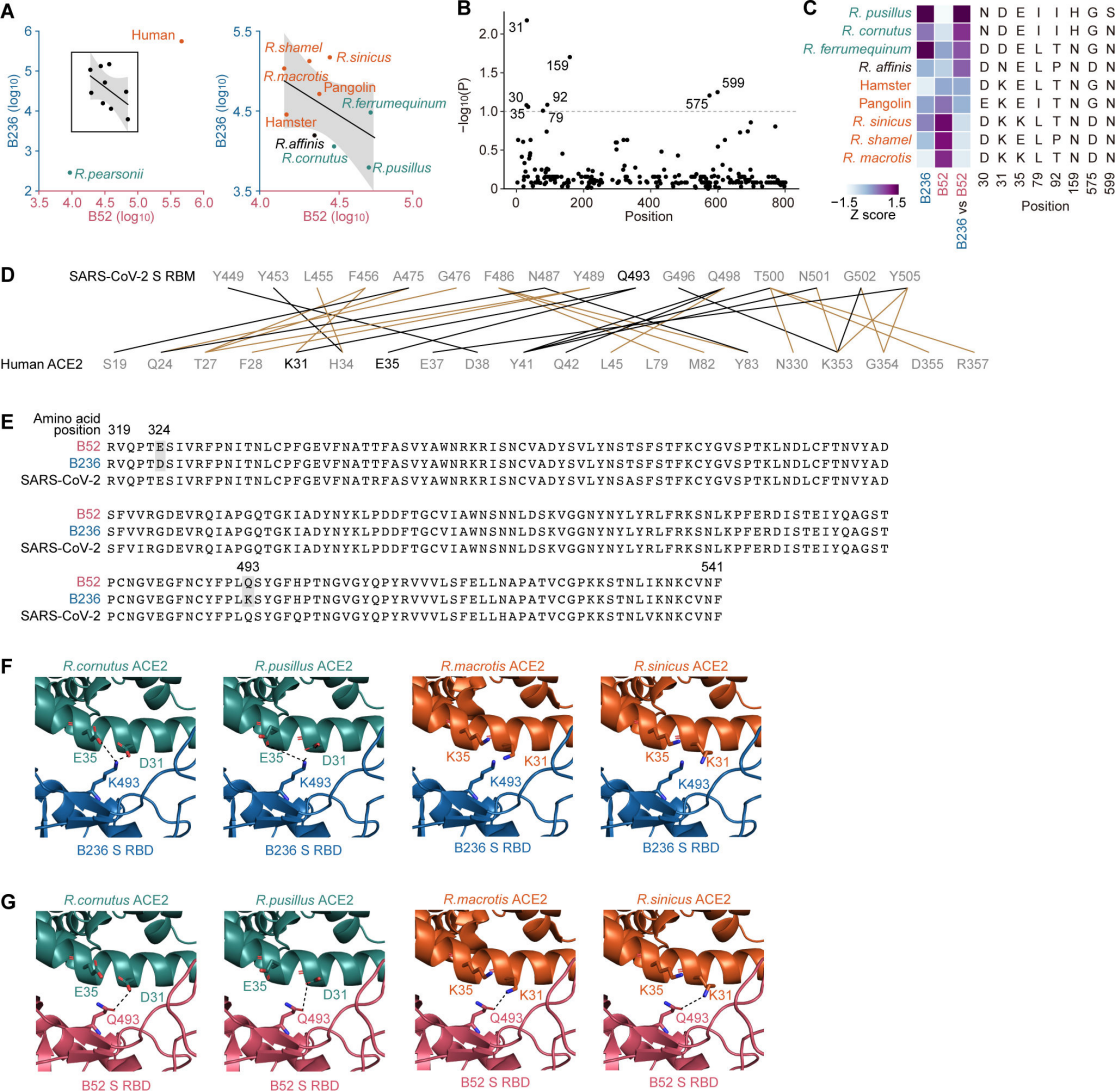
Interaction between *Rhinolophus* bat ACE2 and the S proteins of two Laotian SC2r-CoVs. (**A**) Inverse correlation of the ACE2 susceptibility to B236 and B52 infection. The boxed region is zoomed in on the right panel. (**B**) Association between B52 and B236 infectivity and ACE2 polymorphism among animal species. The association between the relative infectivity [log_10_(B236 infectivity/B52 infectivity)] for each ACE2 protein and each polymorphic amino acid site was evaluated by one-way ANOVA. Dashed line, *P* = 0.1. Outlier species (human and *R. pearsonii*; gray dots in Fig. 2A) were excluded from the analysis. (**C**) Amino acid sites associated with the B236 and B52 infection tropisms. Heatmaps of the Z scores of B236 infectivity, B52 infectivity, and relative infectivity are shown on the left. (D–F) Structural insights into the binding of S RBD and ACE2 proteins. (**D**) The scheme of interaction between the SARS-CoV-2 S receptor binding motif (top) and human ACE2 (bottom). The salt bridge or hydrogen bond is indicated in black, and the van der Waals interaction is indicated in brown. Q493 of SARS-CoV-2 S and K31 and E35 of human ACE2 are indicated in black. This information is referred to in a previous report (17). RBM, receptor binding motif. (**E**) Amino acid alignment of the RBDs of B52 and B236. Residues with nonsynonymous substitutions between B52 and B236 are shaded in gray. The alignment was plotted by Multiple Align Show (https://www.bioinformatics.org/sms/index.html). (**F**) The structural model of the complex of B236 S RBD (blue) and the homology model ACE2 of *R. cornutus* (leftmost, green), *R. pusillus* (the second from the left, green), *R. macrotis* (the second from the right, orange), or *R. sinicus* (rightmost, orange), respectively. The model was reconstructed by using the co-structure of B236 S RBD and human ACE2 (PDB: 7PKI, https://www.rcsb.org/structure/7PKI) (6) as templates and homology models. The residue 493 of B236 S RBD and the residues 31 and 35 of ACE2s are indicated as stick models. Dashed lines indicate salt bridges. (**G**) The structural model of the complex of B52 S RBD (red) and the homology model ACE2 of *R. cornutus* (leftmost, green), *R. pusillus* (the second from the left, green), *R. macrotis* (the second from the right, orange), or *R. sinicus* (rightmost, orange), respectively. The model was reconstructed by using the co-structure of B236 S RBD and human ACE2 (PDB: 7PKI, https://www.rcsb.org/structure/7PKI) (6) as templates and homology models. The residue 493 of B52 S RBD and the residues 31 and 35 of ACE2s are indicated as stick models. Dashed lines indicate hydrogen bonds.

To identify the genetic determinants of susceptibility to B52 and B236 infections in ACE2 proteins, we then assessed the amino acid polymorphisms in ACE2 proteins that can be associated with susceptibility to B52 and B236. As shown in Fig. 2A, the susceptibility of ACE2 proteins to B236 exhibited a strong inverse correlation with that of B52 (except for the ACE2 proteins of humans and *R. pearsonii*). We calculated the relative infectivity score between B236 and B52 [i.e., log_10_(B236 infectivity/B52 infectivity)] for each ACE2 and subsequently evaluated the association between this score and amino acid polymorphism at each site in the ACE2 protein. For the analysis, the data for outlier species, humans and *R. pearsonii*, were excluded. At the permissive statical threshold (*P* < 0.1), we detected eight amino acid sites associated with susceptibility (Fig. 2B and C). Of these, residue 35 exhibited the strongest association (*P* = 0.0067). In these eight residues, four residues positioned at 30, 31, 35, and 79 are located in the region that interacts with the SARS-CoV-2 S RBD (17). Particularly, Shang et al. showed that the residues positioned at 31 (K31) and 35 (E35) of human ACE2 are crucial to form hydrogen bonds with Q493 of SARS-CoV-2 S (Fig. 2D) (17). When we focus on the virus side, the amino acid similarity of the RBDs of B52 and B236 is very high (221/223; 99.1%), and the two amino acid residues positioned at 324 (D324 for B236, E324 for B52) and 493 (K493 for B236, Q493 for B52) are different in the RBD between B52 and B236 (Fig. 2E). Importantly, out of the two residues that differ in the RBDs of B52 and B236, only residue 493 can interact with human ACE2 (Fig. 2D and E). Moreover, the co-crystal structure of B236 S RBD and human ACE2 showed that the K493 of B236 S interacts with the E35 of human ACE2 (6). Because a previous paper (17) and our analyses (Fig. 2A through C) suggested the importance of the residues positioned at 31 and 35 of ACE2 to interact with viral S protein, we addressed the possibility that the residues 31 and 35 of *Rhinolophus* bat ACE2 interact with the residue 493 of B236/52 S. We prepared homology models of B52 S RBD and the ACE2 proteins of four *Rhinolophus* bats, *R. cornutus*, *R. macrotis*, *R. pusillus*, and *R. sinicus*, and replaced the co-structure of B236 S RBD and human ACE2 (PDB: 7PKI) (6) with those models. As summarized in Fig. 2C and Table 1, the ACE2 tropism of B236/52 is closely correlated to the two residues positioned at 31 and 35. In fact, the replaced models of S RBD and ACE2 showed that the K493 of B236 formed salt bridges with the D31 and E35 of the ACE2 proteins of *R. cornutus* and *R. pusillus*, while electrostatic repulsion was observed between the K493 of B236 and the K31 and K35 of the ACE2 proteins of *R. macrotis* and *R. sinicus* (Fig. 2F). In contrast, the Q493 of B52 formed hydrogen bonds with the K31 of the ACE2 proteins of *R. macrotis* and *R. sinicus* or with D31 of the ACE2 proteins of *R. cornutus* and *R. pusillus* (Fig. 2G). These observations suggest that the electrostatic interaction between residue 493 of the B236/52 S protein and residues 31 and 35 of the host ACE2 protein is associated with the tropism of B52 and B236.

**TABLE 1:** Summary of residues 31 and 35 of ACE2 and the tropism of B236 and B52

	Residue		
Bat species of ACE2	31	35	ACE2 tropism of B236 and B52
*R. cornutus*	D	E	B52<B236
*R. pusillus*	D	E	B52<B236
*R. macrotis*	K	K	B52>B236
*R. sinicus*	K	K	B52>B236
*R. shameli*	K	E	B52>B236

### Determination of the different tropisms of B236 and B52

**FIG 3:**
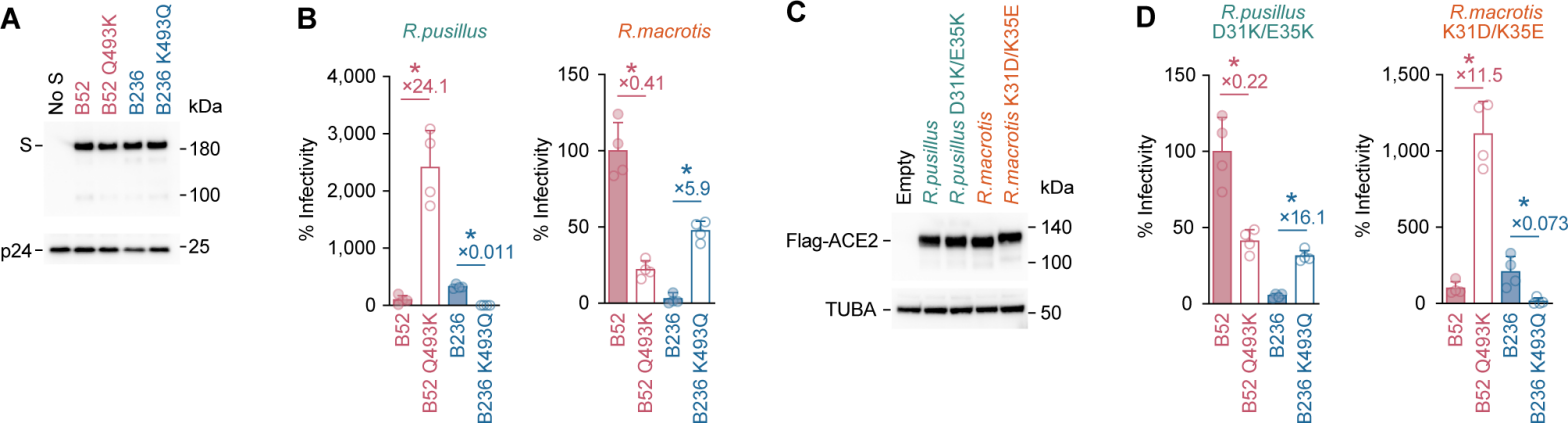
ACE2 tropism is determined by the interaction between residues 31/35 of ACE2 and residue 493 of S. (**A**) Western blotting. A representative blot of pseudovirus is shown. HIV-1 p24 is an internal control for the pseudovirus. kDa, kilodalton. (**B and D**) Pseudovirus assay. HIV-1-based reporter viruses pseudotyped with the S proteins of B236, B52, or their derivatives were prepared. The pseudoviruses were inoculated into a series of HEK293 cells transiently expressing *Rhinolophus* bat ACE2 cells at 2 ng HIV-1 p24 antigen, and the percentages of infectivity compared to that of the virus pseudotyped with B52 are shown. The numbers in the panel indicate the fold change of the B236 value to the B52 value in each target cell. (**C**) Western blotting. A representative blot of the cells transiently expressing flag-tagged *Rhinolophus* bat ACE2 cells is shown. TUBA is an internal control for the cells. kDa, kilodalton. In (**B**) and (**D**),the data are expressed as the mean with SD. Assays were performed in quadruplicate. The numbers in the panel indicate the fold change versus parental S. Statistically significant differences (*P < 0.05) between B236 and B52 were determined by a two-sided Student’s *t*-test.

To address the possibility that the interaction between the residue 493 of viral S protein and the residues 31 and 35 of host ACE2 protein explains the different tropism of B236 and B52, we prepared the two S derivatives of B236 and B52, which harbor the mutations at residue 493: B236 S K493Q and B52 S Q493K. The mutations at residue 493 of S protein did not affect the levels of S proteins incorporated into the released pseudoviral particles (Fig. 3A). We then selected the two *Rhinolophus* ACE2 proteins as representatives: those from *R. pusillus* and *R. macrotis*, which are strongly preferred by B236 and B52, respectively (Fig. 1C), for pseudovirus infection experiments. As shown in Fig. 3B, in the cells expressing *R. pusillus* ACE2, which is preferred by B236, the infectivity of B236 S pseudovirus was significantly decreased (89.7-fold) by the K493Q substitution. In contrast, the infectivity of the B52 S pseudovirus was significantly increased (24.1-fold) by the Q493K substitution (Fig. 3B). In the cells expressing *R. macrotis* ACE2, which is preferred by B52, the infectivity of B52 pseudovirus was significantly decreased (2.4-fold) by the Q493K substitution, while that of B236 was 5.9-fold increased by the K493Q mutation (Fig. 3B). These results suggest that residue 493 of B236/52 S determines the tropism of *Rhinolophus* ACE2.

To further address the possibility that residues 31 and 35 of the ACE2 receptor are responsible for the tropism of B236/52, we generated plasmids expressing *R. pusillus* ACE2 D31K/E35K and *R. macrotis* ACE2 K31D/K35E. The mutations at residues 31 and 35 of *Rhinolophus* ACE2 did not affect their protein expression levels (Fig. 3C). In the case of *R. pusillus* ACE2 D31K/E35K, the B52 infectivity was 4.5-fold decreased by the Q493K substitution, while the B236 infectivity was 16.1-fold increased by the K493Q substitution (Fig. 3D). In the case of *R. macrotis* ACE2 K31D/K35E, the B52 infectivity was 11.5-fold increased by the Q493K substitution, while the B236 infectivity was 13.7-fold decreased by the K493Q substitution (Fig. 3D). Altogether, these findings suggest that the ACE2 tropism of B236 and B52 is determined by residue 493 of viral S proteins, and residues 31 and 35 of ACE2 receptors are responsible for the viral tropism.

## DISCUSSION

In this study, we showed that the SC2r-CoVs identified in Laotian bats, B236 and B52, exhibit different ACE2 tropisms. We further demonstrate that this tropism difference is determined by the amino acid residue positioned at 493 of their S proteins. Structural analysis suggests that residue 493 of the viral S protein plays a critical role in the interaction mediated by a salt bridge with amino acid residues positioned at 31 and 35 of the host ACE2 protein. Our results provide insight into the host tropism of SC2r-CoVs, which is defined by the ACE2 receptor.

Interactions between viral proteins and host receptors that determine host range are known from other viruses [reviewed in reference (18)]. For example, the host tropism of influenza A viruses (IAVs) is determined by the affinity of the viral hemagglutinin for the sialic acids of the host species: human IAVs prefer to bind to α2–3-linked sialic acid, whereas avian IAVs prefer to bind to α2–6-linked sialic acid [reviewed in reference (19, 20)]. In primate lentiviruses (PLVs), including HIV-1, the viral envelope protein binds to two host receptor proteins, the CD4 protein (major receptor) and chemokine receptors (coreceptors), to initiate infection. Russell et al. showed that CD4 receptor diversity is an ancient protective mechanism against PLVs (21). Moreover, although HIV-1 and related primate lentiviruses use CCR5 as the infection coreceptor, some PLVs that are evolutionarily unrelated to HIV-1 use CCR2 (22, 23) or CXCR6 (24 – 26). Furthermore, the host range of the Ebola virus is determined by the difference in amino acid residues in the Niemann-Pick C1 protein, the infection receptor (27, 28). Therefore, the identification of the amino acid residues of the viral S protein that determine the specificity of the host receptor proteins leads to an estimate of the host range of the virus. Furthermore, if the use of human ACE2 and the amino acid residues of the SC2r-CoV S proteins that determine its affinity can be identified, it will be possible to infer from the *S* gene sequence alone whether the SC2r-CoV of concern is capable of spreading to humans.

Li et al. reported on the ACE2 residues crucial for the binding of another SC2r-CoV, RaTG13, to *R. affinis* ACE2 (13). They conducted experiments using seven polymorphic variants of *R. affinis* ACE2 and demonstrated that residues 34 and 38 of ACE2 play a critical role in the binding to RaTG13. Additionally, they generated B52 and B236 pseudoviruses and conducted infection experiments with the polymorphic variants, illustrating that B52 and B236 efficiently infected all *R. affinis* ACE2 variants. These findings are consistent with our results, pointing out that both B52 and B236 exhibit similar infectivity to *R. affinis* ACE2 (Fig. 1C). Based on these results, B52 and B236 are better adapted to *R. affinis* ACE2, including its polymorphic variants, than RaTG13. In our study, employing ACE2 from various *Rhinolophus* species other than *R. affinis*, we observed differences in the bat ACE2 tropisms of B52 and B236. Notably, ACE2 residues 31 and 35 influenced susceptibility (Fig. 3D).

The mutation pattern of residue 493 of SARS-CoV-2 S is extraordinary: the S proteins of prior SARS-CoV-2 variants of concern (VOCs), including the ancestral Wuhan-Hu-1 strain, harbor Q493 (29), while those of the two major SARS-CoV-2 VOCs, Omicron BA.1 and BA.2, harbor R493 (30). Interestingly, the R493 has reverted to Q493 in subsequent Omicron subvariants such as Omicron BA.4, BA.5, BA.2.75, and BQ.1.1 (31 – 34). Regarding this, Wang et al. showed that the R493Q reversion mutation improves the affinity for human ACE2 (35). Additionally, we have previously shown that the R493Q mutation contributes to the evasion of humoral immunity induced by BA.2, which carries R493, in rodents (31). Therefore, residue 493 of SC2r-CoVs may be relatively easily converted to R493 and Q493, depending on immune evasion and/or affinity to the host ACE2 protein, and the humoral immunity of *Rhinolophus* bats against SC2r-CoVs may be associated with the conversion of residue 493.

Here, we focused on two Laotian SC2r-CoVs, B236 and B52. However, there are still limitations to this study. First, as the *ACE2* genes of their host *Rhinolophus* bats, *R. marshalli* and *R. malayanus*, have not yet been determined, we could not test the receptor tropism of these two SC2r-CoVs on their host species. As there are still several *Rhinolophus* bat species in Southeast Asia, it would be possible to map the range and diversity of each SC2r-CoV by studying its habitat and ACE2 gene diversity. Second, although we used the publicly available *ACE2* gene sequences from each *Rhinolophus* bat, previous studies focusing on the *ACE2* genes of *R. affinis* and *R. sinicus* showed the single nucleotide polymorphisms of *ACE2* genes in these species and demonstrated that the allelic variation impacts the susceptibility to SC2r-CoVs (e.g., RaTG13) (13, 15). Therefore, not only the difference in the *ACE2* gene between species, on which we focused this study, but also the single nucleotide polymorphism of the *ACE2* gene within species may affect the tropism of SC2r-CoVs in *Rhinolophus* bats. Third, here we focused on ACE2, the receptor for the infection of SARS-CoV and SARS-CoV-2; however, recent studies by Guo et al. showed that certain SC2r-CoVs infect cells in an ACE2-independent manner (6, 36, 37). Therefore, it would be difficult to exclude the possibility of the existence of alternative receptor(s) for SC2r-CoVs beyond ACE2. Moreover, although most Merbecoviruses, including the Middle East respiratory syndrome coronavirus, use dipeptidyl peptidase 4 for the infection receptor, a report showed that two Merbecoviruses, PDF-2180 and NeoCoV, identified in *Pipistrellus hesperus* bats and *Neoromicia capensis* bats, respectively, employ ACE2 instead of dipeptidyl peptidase 4 for the infection receptor (38). Therefore, coronaviruses may relatively easily change the receptors for infection.

In conclusion, here we showed that *ACE2* genes are diversified in *Rhinolophus* bats and this may determine the tropism of SC2r-CoVs in *Rhinolophus* bat species. The difference in SC2r-CoVs in *Rhinolophus* ACE2 tropism may be a driving force that promotes the diversity of circulating viruses in *Rhinolophus* bats and further confers infectivity to a variety of host species, including humans. Previous studies focusing on the SARS-CoV-2 VOCs have shown that the host range can be altered by mutations in the *S* gene. For example, although the S protein of the ancestral Wuhan-Hu-1 strain is unable to use murine ACE2 as an infection receptor, the N501Y mutation present in the S proteins of the Alpha and subsequent variants allows murine ACE2 to be used for infection (39). Identifying mutations in the S protein that determine host receptor usage should be an important study to estimate the host range of SC2r-CoVs of concern. Further investigations will be needed to identify and predict SC2r-CoVs in the wild that can be transmitted to humans.

## MATERIALS AND METHODS

### Nucleotide sequence data collection

The whole genome sequences of known SC2r-CoVs and three SC1r-CoVs [SARS-CoV-1 Tor2 (NC_004718.3), WIV1 (KF367457.1), and LYRa11 (KF569996.1)] were downloaded from NCBI GenBank (https://www.ncbi.nlm.nih.gov/; download date: 24 March 2023). Also, the nucleotide sequences of ACE2 from various species were collected from NCBI GenBank (download date: 24 March 2023). Information on the sarbecoviruses and ACE2 sequences is summarized in Tables 2 and 3.

**TABLE 2:** ACE2 sequences used in this study

Host species	Accession no.
*Rhinolophus affinis*	MT394225.1
*Rhinolophus cornutus*	LC564973
*Rhinolophus ferrumequinum*	AB297479.1
*Rhinolophus macrotis*	GQ999932.1
*Rhinolophus pearsonii*	EF569964.1
*Rhinolophus pusillus*	GQ999938.1
*Rhinolophus shameli*	MZ851782.1
*Rhinolophus sinicus*	KC881004.1
Hamster	XM_005074209.3
Human	NM_021804.3
Pangolin	XM_017650263.2

**TABLE 3:** Sarbecovirus sequences used in this study

Virus	Accession no.
BANAL-20-236	MZ937003.2
BANAL-20-103	MZ937001.1
BANAL-20-52	MZ937000.1
Wuhan-Hu-1	NC_045512.2
MP789 (MpCoV-GD)	MT121216.1
Guangdong/1/2019	EPI_ISL_410721
RShSTT200	EPI_ISL_852605
RShSTT182	EPI_ISL_852604
RaTG13	MN996532.2
GX-P2V	MT072864.1
GX-P5L	MT040335.1
GX-P4L	MT040333.1
GX-P1E (MpCoV-GX)	MT040334.1
GX-P5E	MT040336.1
Rc-kw8	LC663793.1
Rc-mk2	LC663959.1
Rc-o319	LC556375.1
Rc-os20	LC663958.1
PDF-2370	MT726044.1
PDF-2386	MT726043.1
PRD-0038	MT726045.1
BtKY72	KY352407.1
BM48-31	NC_014470.1
Khosta-1	MZ190137.1
Khosta-2	MZ190138.1
RhGB01	MW719567.1
Tor2	NC_004718.3
WIV1	KF367457.1
LYRa11	KF569996.1

### Molecular phylogenetic analysis

The ML tree of RBDs of SC2r-CoVs was constructed by the following procedures: multiple sequence alignment (MSA) was constructed using MAFFT v7.511 (40) with the default option. Alignment sites with <30% site coverage were excluded using trimAl v1.2rev59 (41). Alignment sites corresponding to the RBD of SARS-CoV-2 S (e.g., nucleotide positions 22,517–23,185 in the SARS-CoV-2 Wuhan-Hu-1 strain) were used for the tree construction. The ML tree was constructed using RAxML-NG v1.1.0 (42) under the GTR + G + I nucleotide substitution model with 100 bootstrap analyses.

In Fig. 1E, the ML tree of nucleotide sequences of *Rhinolophus* ACE2 was constructed by the following procedures: MSA was constructed using MUSCLE (43) implemented in MEGA11 v11.0.13 (44). The ML tree was constructed using the ML method implemented in MEGA11 under the T92 + G substitution model with 100 bootstrap analyses.

### Cell culture

HEK293 cells (a human embryonic kidney cell line; ATCC, CRL-1573), HEK293T cells (a human embryonic kidney cell line; ATCC, CRL-3216), and HOS-ACE2/TMPRSS2 cells (a human osteosarcoma cell line; ATCC, CRL-1543) stably expressing human ACE2 and TMPRSS2 (45, 46) were maintained in Dulbecco’s modified Eagle’s medium (high glucose) (Sigma-Aldrich, Cat# 6429-500ML) containing 10% fetal bovine serum and 1% penicillin-streptomycin (Sigma-Aldrich, Cat# P4333-100ML). The HOS-TMPRSS2 cells that stably express *Rhinolophus* bat ACE2 were generated as described below (see the section Generation of HOS-TMPRSS2 cells stably expressing a variety of ACE2 proteins) and maintained in Dulbecco’s modified Eagle’s medium (high glucose) (Sigma-Aldrich, Cat# 6429-500ML) containing 10% fetal bovine serum and 1% penicillin-streptomycin (Sigma-Aldrich, Cat# P4333-100ML).

### Plasmid construction

Plasmids expressing the codon-optimized S proteins of B236 (GenBank accession no. MZ937003.2) and B52 (GenBank accession no. MZ937000.1) were synthesized by a gene synthesis service (Fasmac). Plasmids expressing the ACE2 proteins of *R. sinicus* (GenBank accession no. KC881004.1), *R. ferrumequinum* (GenBank accession no. AB297479.1), *R. shameli* (GenBank accession no. MZ851782.1), *R. pearsonii* (GenBank accession no. EF569964.1), *R. pusillus* (GenBank accession no. GQ999938.1), *R. macrotis* (GenBank accession no. GQ999932.1), *R. affinis* (GenBank accession no. MT394225.1), hamster (GenBank accession no. XM_005074209.3), and pangolin (GenBank accession no. XM_017650263.2) were also synthesized by a gene synthesis service (Fasmac). For *R. cornutus* ACE2 (GenBank accession no. LC564973.1), pCAGGS-blast-RcACE2 (47) was provided. Plasmids expressing the derivatives of codon-optimized S proteins of B236 and B52 and *Rhinolophus* bat ACE2 were generated by site-directed overlap extension PCR using the following primers:

BANAL52 S-Fw, 5′-cta tag ggc gaa ttg ggt acc atg ttc gtc ttc ctc-3′; BANAL52 S-Rv, 5′-agc tcc acc gcg gtg gcg gcc gct cat gtg tag tgc aa-3′; BANAL236 S-Fw, 5′-cta tag ggc gaa ttg ggt acc atg ttg ttc ttc ttc-3′; BANAL236 S-Rv, 5′-agc tcc acc gcg gtg gcg gcc gct cat gtg tag tgg ag-3′; BANAL-52-Q493K-Fw, 5′-gtt att ttc cac tta agt cat acg gat tcc-3′; BANAL-52-Q493K-Rv, 5′-gga atc cgt atg act taa gtg gaa aat aac-3′; BANAL-236-K493Q-Fw, 5′-gtt act tcc cac tgc aat cat atg gat tcc-3′; BANAL-236-K493Q-Rv, 5′-gga atc cat atg att gca gtg gga agt aac-3′; R_pusillus_inf-Fw, 5′-cta gcc tcg agg ttt gga tcc gcc acc atg tca ggc-3′; ACE2_univr_inf-Rv, 5′-agt tta aac act agt acg cgt cta ctt gtc atc gtc-3′; R_macrotis_inf-Fw, 5′-cta gcc tcg agg ttt gga tcc gcc acc atg tca ggc-3′; R_pusillus_D31K_E35K-Fw, 5′-aaa ttt ttg aac aag ttt aac tcc aaa gct gaa gac ctg-3′; R_pusillus_D31K_E35K-Rv, 5′-cag gtc ttc agc ttt gga gtt aaa ctt gtt caa aaa ttt-3′; R_macrotis_K31D_K35E-Fw, 5′-aaa ttt ttg gac gac ttt aac tct gaa gct gaa gac ctg-3′; R_macrotis_K31D_K35E-Rv, 5′-cag gtc ttc agc ttc aga gtt aaa gtc gtc caa aaa ttt-3′. The resulting PCR fragment was cloned into the KpnI-NotI site of the backbone pCAGGS vector (48) (for the S expression plasmids) or the BamHI/MluI site of pWPI-ACE2-zeo (for ACE2 expression plasmids) (46) with a 3× FLAG tag at the C-terminus using the In-Fusion HD Cloning Kit (Takara, Cat# Z9650N). Nucleotide sequences were determined by DNA sequencing services (Eurofins), and the sequence data were analyzed by Sequencher v5.1 software (Gene Codes Corporation).

### Generation of HOS-TMPRSS2 cells stably expressing a variety of ACE2 proteins

To prepare lentiviral vectors expressing ACE2, HEK293T cells (2,000,000 cells) were cotransfected with 12 µg of pCAG-HIVgp, 10 µg of pCMV-VSV-G-RSV-Rev, and 17 µg of either pWPI-ACE2-zeo by the calcium phosphate method. After 12 h of transfection, the culture medium was changed to fresh medium. After 48 h of transfection, the culture supernatant, including lentivector particles, was collected. HOS-TMPRSS2 cells (100,000 cells) were then transduced with the ACE2-expressing lentiviral vector. After 48 h post transduction, transduced cells were maintained for zeocin (50 µg/mL; Invivogen, Cat#ant-zn-1) selections for 14 days.

### Pseudovirus assay

A pseudovirus assay was performed as previously described (29 – 32, 46, 49 – 57). Briefly, HIV-1-based, luciferase-expressing reporter viruses were pseudotyped with the S proteins of B236, B52, and their derivatives. HEK293T cells (3,000,000 cells) were cotransfected with 4 µg psPAX2-IN/HiBiT (58), 4 µg pWPI-Luc2 (58), and 2 µg plasmids expressing parental S or its derivatives using PEI Max (Polysciences, Cat# 24765-1) according to the manufacturer’s protocol. Two days post transfection, the culture supernatants were harvested, and the pseudoviruses were stored at −80°C until use. For pseudovirus infection, the amount of input virus was normalized to the HiBiT value measured by the Nano Glo HiBiT lytic detection system (Promega, Cat# N3040), which indicates the amount of p24 HIV-1 antigen. For target cells, the HOS-TMPRSS2 cells stably expressing a variety of *Rhinolophus* bat ACE2 (Fig. 1D) and the HEK293 cells transfected with the plasmids expressing *R. pusillus* and *R. macrotis* ACE2 and their derivatives with TransIT-LT1 (Takara, Cat# MIR2300) (Fig. 3B and D) were used. Two days post infection, the infected cells were lysed with a Bright-Glo Luciferase Assay System (Promega, Cat# E2620), and the luminescent signal was measured using a GloMax Explorer Multimode Microplate Reader (Promega).

### Association analysis between the B236 and B52 infection tropisms and polymorphic sites in ACE2 among animals

The results of the B236 and B52 pseudoviral infection assays in cells expressing ACE2 from *Rhinolophus* bats and other representative species were used. Of these, results for *R. affinis*, *R. cornutus*, *R. ferrumequinum*, *R. macrotis*, *R. pusillus*, *R. shameli*, *R. sinicus*, hamster, and pangolin were used. We excluded the data for humans and *R. pearsonii* from the analysis because the data for these species deviated from the trend in which ACE2 proteins more sensitive to B236 infection are less sensitive to B52. First, we calculated relative infectivity [log_10_(B236 infectivity/B52 infectivity)] for each ACE2 protein. This relative infectivity score was used as an objective variable for the association analysis. We constructed the MSA of ACE2 amino acid sequences using MAFFT v7.511 (40) with the default option. We used amino acid residues in each alignment site of the MSA as qualitative explanatory variables. The statistical significance of the association between the relative infectivity and amino acid residues in an ACE2 polymorphic site was evaluated using a one-way ANOVA. The analysis was performed in R v4.2.1.

### Protein structure model

All protein structural analyses were performed using Discovery Studio 2021 (Dassault Systèmes BIOVIA). In Fig. 2F and G, the crystal co-structure of B236 S RBD and human ACE2 (PDB: 7PKI, https://www.rcsb.org/structure/7PKI) (6) was used as the template, and 40 homology models of the B52 S RBD were generated using the Build homology model protocol MODELLER v9.24 (59). Evaluation of the homology models was performed using PDF total scores and DOPE scores, and the best model for the B52 S was selected. Homology models of ACE2 in *R. cornutus*, *R. pusillus*, *R. macrotis*, or *R. sinicus* were generated in the same way as B52 S RBD. The crystal co-structures of B236 S RBD and human ACE2 (PDB: 7PKI, https://www.rcsb.org/structure/7PKI) (6) were used. To predict interaction between S RBD and ACE2, the structure of human ACE2 was replaced by the homology models of ACE2 in *R. cornutus*, *R. pusillus*, *R. macrotis*, or *R. sinicus*. In Fig. 2G, the structure of B236 S RBD was replaced by the homology model of B52 S RBD.

### Western blot

Western blot was performed as previously described (30, 31, 52, 54, 56, 60). For the blot, the supernatants of HEK293T cells cotransfected with the S expression plasmids and HIV-1-based pseudovirus producing plasmids, or HEK293 cells cotransfected with bat ACE2 expression plasmids (see the section Pseudovirus assay) were used. The harvested cells were washed and lysed in RIPA buffer [50  mM Tris-HCl buffer (pH 7.6), 150  mM NaCl, 1% Nonidet P-40, 0.5% sodium deoxycholate, 0.1% SDS], protease inhibitor cocktail (Nacalai Tesque, Cat# 03969-21). The lysates were diluted with 2× sample buffer [100 mM Tris-HCl (pH 6.8), 4% SDS, 12% β-mercaptoethanol, 20% glycerol, 0.05% bromophenol blue]. Both samples were boiled for 10 m. Then, 10 µL samples were subjected to Western blotting. In addition, 900 µL culture medium containing the pseudoviruses at 250 ng HIV-1 p24 antigen was layered onto 500 µL 20% sucrose in PBS and centrifuged at 20,000 × *g* for 2 h at 4°C. Pelleted virions were resuspended in 1× sample buffer [50 mM Tris-HCl (pH 6.8), 2% SDS, 6% β-mercaptoethanol, 10% glycerol, 0.0025% bromophenol blue] and boiled for 10 m. For protein detection, the following antibodies were used: mouse anti-SARS-CoV-2 S monoclonal antibody (clone 1A9, GeneTex, Cat# GTX632604, 1:10,000), mouse anti-HIV-1 p24 monoclonal antibody (183-H12-5C, obtained from the HIV Reagent Program, NIH, Cat# ARP-3537, 1:1,000), mouse anti-alpha-tubulin (TUBA) monoclonal antibody (clone DM1A, Sigma-Aldrich, Cat# T9026, 1:10,000), horseradish peroxidase (HRP)-conjugated mouse anti-FLAG monoclonal antibody (clone M2, Sigma-Aldrich, Cat# A8592, 1:1,000), and HRP-conjugated horse anti-mouse IgG antibody (Cell Signaling, Cat# 7076S, 1:2,000). Chemiluminescence was detected using SuperSignal West Femto Maximum Sensitivity Substrate (Thermo Fisher Scientific, Cat# 34095) or Western Lightning Plus-ECL (PerkinElmer, Cat# NEL104001EA) according to the manufacturer’s instructions. Bands were visualized using the ChemiDoc Touch Imaging System (Bio-Rad).

## Data Availability

The computational codes used in the present study are available on the GitHub repository (https://github.com/TheSatoLab/BANAL_tropism).
